# Aneurysmal Subarachnoid Hemorrhage Models: Do They Need a Fix?

**DOI:** 10.1155/2013/615154

**Published:** 2013-06-26

**Authors:** Fatima A. Sehba, Ryszard M. Pluta

**Affiliations:** ^1^Departments of Neurosurgery and Neuroscience, Mount Sinai School of Medicine, New York, NY 10029, USA; ^2^Surgical Neurology Branch, National Institute of Neurological Disorders and Stroke, National Institutes of Health, Bethesda, MD 20892, USA

## Abstract

The discovery of tissue plasminogen activator to treat acute stroke is a success story of research on preventing brain injury following transient cerebral ischemia (TGI). That this discovery depended upon development of embolic animal model reiterates that proper stroke modeling is the key to develop new treatments. In contrast to TGI, despite extensive research, prevention or treatment of brain injury following aneurysmal subarachnoid hemorrhage (aSAH) has not been achieved. A lack of adequate aSAH disease model may have contributed to this failure. TGI is an important component of aSAH and shares mechanism of injury with it. We hypothesized that modifying aSAH model using experience acquired from TGI modeling may facilitate development of treatment for aSAH and its complications. This review focuses on similarities and dissimilarities between TGI and aSAH, discusses the existing TGI and aSAH animal models, and presents a modified aSAH model which effectively mimics the disease and has a potential of becoming a better resource for studying the brain injury mechanisms and developing a treatment.

## 1. Introduction

Stroke is the second major cause of death worldwide. According to the World Stroke Organization approximately 15 million people suffer from stroke each year. Five million people die from it, and another 5 million are left permanently disabled [1]. Ischemic stroke constitutes the most and hemorrhagic stroke 15 to 30% of the total annual stroke cases [2]. The 21-day to 1-month case fatality ranges from 13 to 23% for ischemic stroke, as compared to 25–35% for hemorrhage stroke [3]. The cost of survivor care and lost productivity (conservatively estimated to be more than 54 billion dollars annually) necessitates research to reduce stroke mortality and disability. An essential step in this direction is developing an experimental model that replicates the human condition. Numerous animal models addressing causes and pathophysiology of ischemic and hemorrhagic stroke have been developed. Whereas research using these models has clearly influenced the treatment of global ischemic stroke, it has made relatively small impact on treatment of hemorrhagic stroke.

## 2. Ischemic and Hemorrhagic Brain Injury

Ischemic stroke occurs when blood supply to the brain is reduced to a level that cerebral function and metabolism are no longer maintained. Cerebral ischemia could be focal or global and transient or permanent. A mix of any of them is also possible; for instance, after aneurysmal subarachnoid hemorrhage, a patient can develop a transient global ischemia (evoked by temporary increased ICP) followed by permanent focal ischemia because of a thrombosis or delayed vasospasm. Transient focal ischemia (TFI) affects a specific brain region, and transient global ischemia (TGI) affects the whole brain for a limited time; both are followed by reperfusion and/or hyperperfusion. In contrast to transient ischemia, in permanent ischemia blood flow is never reestablished to the part (local) or the whole (global) brain. Hemorrhagic stroke occurs when blood flow in the brain is reduced due to the intracranial bleeding. Aneurysmal subarachnoid hemorrhage (aSAH), a nontraumatic type of the intradural bleeding, constitutes 5% of all strokes and occurs when an intracranial aneurysm bursts and spews blood under high pressure into the subarachnoid space. Such a violent flow of blood into a narrow, CSF-filled space results in a dramatic increase in intracranial pressure and decrease in the cerebral perfusion pressure (CPP) and cerebral blood flow (CBF) [4–6]. The ICP-dependent reduction in CBF after SAH is beneficial but also harmful, beneficial as it saves a patient's life by allowing a blood clot to seal the dome of the ruptured aneurysm and stop the bleeding and harmful as it limits blood flow to the whole brain for unpredictable time and may result, in the best-case scenario, in a transient local or global ischemic brain injury or, in the worst case, brain death. Thus, to best of our knowledge most of aSAH bleeding is associated with transient global hypoperfusion and/or ischemia. Though a role of TGI in aSAH outcome has been suspected since early 19th century, its true nature remains poorly defined and its importance largely unappreciated. As a consequence, the differences and similarities between TGI and aSAH are not determined, and knowledge that TGI researchers have accumulated over the years is not used to further understanding of the SAH-related injury to the brain. Three reasons of this oversight are: (1) events leading to a “spontaneous” TGI and TGI evoked by an aneurysmal SAH are different, (2) the sudden/abrupt nature of aSAH event makes association with TGI difficult to study [5, 7], and (3) until recently, most research on improving patient outcome has been on delayed cerebral vasospasm (AKA delayed neurological deficits) and not on events that occur early after aSAH [8]. Lately, limited improvement in patient outcome after more than half a century of research convinced many researchers to reevaluate the significance of early events and more importantly the influence of early TGI after aSAH on the outcome [9].

In recognition of this new trend we review animal models of TGI and aSAH, discuss why ischemic, but not aSAH models have proven successful in reducing the death and disability after stroke, and propose a modified aSAH model that incorporates features of TGI model and could be a better resource for studying the injury mechanisms and treatment of aSAH.

## 3. Animal Models of TGI and SAH

### 3.1. TGI Models (Table 1)

Animal models of TGI induce either complete or incomplete global ischemia. In complete global ischemia blood flow is ceased completely, and in the incomplete global ischemia blood flow decreases to a degree that cellular metabolism and function can no longer be maintained [49, 50]. The injury and survival are proportional to the duration of global ischemic insult: greater when the insult is a short and resolvable; lasting 10 to 30 minutes and lower when the insult is longer or permanent. Thus, permanent TGI models work best for studying the mechanisms of injury, and the resolvable TGI models work best for studying therapeutic interventions. Below we describe the most extensively used TGI models. See Table 1 for a list of TGI models and animal species used.

#### 3.1.1. Two-Vessel Occlusion (2-VO) Model

Ischemia in this model is created by a transient bilateral carotid occlusion. Variations that allow investigator to control injury intensity are available. A mild-to-moderate injury is created by keeping arterial blood pressure normal during carotid occlusion [40, 51]. A severe injury is achieved by reducing arterial blood pressure to 40–50 mmHg during carotid occlusion. Blood pressure reduction is achieved by phlebotomy or by pharmacological manipulation [52].

The advantages of 2-VO model include one-stage surgical preparation, production of high-grade forebrain ischemia, ability to control ventilation to ensure normoxia and normocarbia, ease of reestablishing cerebral circulation, suitability for chronic studies, and a relatively low failure rate. The disadvantage is that pharmacologically induced hypotension may complicate the interpretation of results [78].

#### 3.1.2. Four-Vessel Occlusion Model(4-VO)

Ischemia in this model is created by almost simultaneous occlusion of four major cerebral vessels: bilateral both vertebral and common carotid arteries [43]. Usually, first, the vertebral arteries are electrocoagulated, and then the common carotid arteries are occluded by tightening the ligatures around them [78]. This model has been extensively studied to assure a high incidence of successful ischemia with acceptable mortality rate. Nevertheless, even in the best hands, animal survival rate following 4-VO is only 50% [43, 79]. A modification of 4-VO which combines a mild systemic hypotension (80–90 mmHg) with bilateral carotid occlusion creates less morbidity and more uniform brain injury [80, 81].

Both 2- and 4-VO models are frequently used to study TGI (see Table 1). 2-VO model is often preferred over 4-VO model as it requires less surgical manipulations; 4-VO requires two state surgical preparation and rarely achieves complete reversal of global ischemia [82]. 

#### 3.1.3. Bihemispheric Forebrain Compression Ischemia (BFCI)

This model was developed by Kramer and Tuynman in 1967 to define the duration of ischemia tolerated by the brain [28]. Ischemia here is induced by increasing intracranial pressure to the level of systolic blood pressure so that cerebral perfusion is disrupted. The increase in intracranial pressure is achieved by infusion of artificial cerebrospinal fluid (CSF) into the cisterna magna. Cushing's reflex evoked by increased ICP can be reduced by administration of the ganglion-blocking drug [83].

TGI produced by BFCI is consistent, reproducible and successfully created in several animal species. Though BFCI model is not as extensively used as the 2-VO or 4-VO model, it provides an excellent foundation for the modified aSAH model that we later propose in this review (see below). 

### 3.2. SAH Models (Table 2)

Brain injury evoked by aSAH consists of early and delayed events. Early events include rise in ICP, fall in CBF and CPP at the time of aSAH, and the delayed events are arterial vasospasm and delayed ischemic deficits that develop 3–7 days after the initial bleed. Due to unpredictable nature (not every aneurysm ruptures) of aSAH [5, 7], the information on ultra-early events is available only as the patient is admitted and monitored after the initial aneurysm rupture. Consequently, information obtained is already delayed, unless rebleed occurs, usually within hours after the initial bleed. However, because of the lingering effects of the initial bleed, the data obtained during rebleed cannot be directly extrapolated as a mimic of the first aSAH [5, 7]. Nevertheless, information obtained during the rebleed has been used to develop animal models of aSAH. These models are widely used to study early and delayed brain injury after aSAH and are accepted as mimics of clinical aSAH (see Table 2 for details) [84–86]. aSAH models can be broadly divided into three categories.

#### 3.2.1. Blood or Hemolysate Injection or Infusion

Injection model involves introduction of autologous fresh blood [56, 67, 74, 87–89] into the cisterna magna, prechiasmatic cistern [90], or next to an intracranial [91, 92] or an extracranial artery [84, 93–97]. This model is quite extensively used to study early and delayed injury after aSAH. In several species (mouse, rat, and dog), a second blood injection 24 to 48 hours after the first is necessary for development of delayed vasospasm. Advantages of this model are that it produces reproducible injury and allows use of saline injected sham control. Disadvantage is a failure to reproduce the mechanical trauma, the first insult felt by the cerebral vasculature upon aneurysm rupture (for review see [98] and references within).

#### 3.2.2. Blood Clot Placement

In this model arterial blood is withdrawn and allowed to clot *ex vivo* and then surgically placed on the adventitial surface of an artery. Both intracranial (the middle cerebral artery [75]) and extracranial (femoral [96]) arteries have been used for clot placement. This model studies delayed vasospasm and not early injury. Advantages of this technique are the well-defined course of vasospasm and low animal mortality that permits pharmacological intervention. Disadvantages are lack of reproducing mechanical trauma (see above) and the high cost of experiment; this model is predominantly used in larger animals: dog, pig, and monkey.

#### 3.2.3. Arterial Puncture

This aSAH model involves puncture of the intracranial artery adjacent to the skull base by an endovascular filament. The model is considered the best mimic of human aSAH as it replicates the mechanical trauma felt by cerebral vasculature upon aneurysm rupture, as well as the events observed during rebleed in aSAH patients: rapid fall in cerebral blood flow and blood accumulation into subarachnoid space [4–6, 98]. However, due to a number of reasons explained elsewhere this model provides a poor control of bleeding and high mortality (for review see [98–100]). Other disadvantages include complicated surgical procedure that requires a trained person and difficultly in adaptation to other, larger species. Nevertheless, arterial puncture is frequently used to study early injury after aSAH especially in rodents.

## 4. Success of Embolic Ischemia Model and Lesson Learnt about aSAH

The research focused on treatment of cerebral ischemia has been successful. It has provided us with recombinant tissue plasminogen activator (r-tPA) that, when used within 4.5 hours after ischemic episode, reduces brain injury and improves the outcome [101]. In contrast, despite extensive research, a therapy that could be translated to clinical SAH has not been found. Though several compounds have been found promising against SAH in animals, none succeed in clinical trials [98]. 

A proper disease modeling may have contributed to the success of TGI research. That varying degree and duration of CBF reduction produce varying effects on the neurovascular unit has been realized [102], and animal models that address a specific problem are accordingly developed. Focal ischemia models study injury following a thrombotic event, and global ischemia models study injury following cardiac arrest. Both models focus on developing a time-dependent intervention. Animal species used range from rodents to the AHA recommended primates [103, 104]. However, even this meticulous approach has not always worked. An example of failure is the free radical-trapping agent NXY-059 that showed promise as a neuroprotectant in rat and primate ischemic models but was ineffective in patients [105]. On the other hand, a spectacular success was the development of thrombolytic therapy with recombinant tissue plasminogen activator (rtPA) against acute transient focal and global ischemic stroke based on the results of studies using a rabbit model of embolic stroke [106, 107]. The success of a rabbit embolic model versus failure of favored ischemic primate model in development of successful treatment may indicate that an accurate model of a disease should provide results that are reproduced across species and successfully translated to clinic. 

## 5. aSAH Models and Components of Injury

Mortality, neurological deficits, and diminished quality of life are the most important end points of a brain injury evoked by TGI and aSAH. However, some and not all of the mechanisms that TGI and aSAH evoke are shared (see Table 3). For example fall in CBF creating temporary global perfusion deficits occurs both in TGI and aSAH, but injury by a prolonged presence of blood in the subarachnoid space characterizes SAH only. Thus, in a new desired aSAH model all components of injury, the presence of controllable TGI, and an intracranial bleeding need to occur simultaneously. Unfortunately, the current animal models dissociate TGI from aSAH, replicate subarachnoid bleed but not a perfusion deficit that creates TGI, and thus these models only partially imitate injury produced by aSAH. This shortcoming may have contributed to a lack of clinical translation of therapies found successful in animals. A more inclusive model that incorporates all components of brain injury after aSAH is required to accelerate the development of adequate treatment for improving the patient's outcome. 

## 6. A Modified aSAH Model

A number of different aSAH models are available for studying injury mechanism and treatment. Each carries its own advantages and disadvantages. One shortcoming common at all is the lack of requirement of CPP fall at SAH induction to a level that ensures TGI. As a result these models replicate some but not all of the components of injury that are present in human aSAH (discussed above). We here propose a modified aSAH model that reproduces all of the components of injury after aSAH and in addition requires limited surgical manipulation, carries low mortality, can be easily adapted to a number of species, and makes comparisons and interpretation of data from different laboratories possible.

After reviewing the existing aSAH models (above) we have formed an opinion that perhaps an *adaptation *of Kramer and Tuynman's TGI model (explained above), that uses autologous arterial blood instead of artificial CSF, provides the best foundation for the modified aSAH model [28]. 

Below we detail three features essential to this modified aSAH model. We discuss the reason we consider them essential and the techniques that can be used to attain them.

### 6.1. Blood Injection

As blood upon aneurysm rupture is released under high pressure and pools into subarachnoid cisterns, a proper replication requires the same to occur in the animal model. The location, speed, and volume blood injection all are important considerations for consistent replication of aSAH injury in animals used within an experiment and across laboratories. 

#### 6.1.1. Blood Delivery Route

Technical details of each procedure can be found in adequate reference(s) in Table 2.

Several routes have been successfully used for intracranial, subarachnoid injection/infusion of blood. A brief description of these routes and techniques is presented below. Details can be found in the references in Table 2.

*A Percutaneous Delivery of Blood.* This route is often favored in small and large animals (rabbit, cat, dog, pig, and monkey). This technique requires good anatomical knowledge and reasonable but basic surgical skills. Briefly, after proper anesthesia and skin preparation (includes shaving the back of the head, between the ears, and the flexor surface of the neck), a short bevel 25-/27-gauge needle attached to an insulin syringe (size depends on the species used, can range from insulin to 10 cc) is introduced in the midline directly below the palpable edge of the cranium after significant head flexion. The needle is slowly advanced until an access to cisterna magna is confirmed with CSF presence in the syringe. At this moment a syringe is exchanged for the one that is filled with fresh, arterial, and preferably nonheparinized blood. Blood is then quickly (in less than 1 min) injected into subarachnoid space. The volume, time, and speed of injection are guided by the rise in ICP to mean arterial blood pressure rendering CPP zero or a drop in CBF below 10 mL/100 g/min. At this moment the needle is quickly removed, and steady compression is applied to the neck. The animal's head is then either returned to neutral or slightly extended position with the body of animal (if possible) tilted down for about 5–10 min to allow blood to flow toward the anterior cisterns. Monitoring of ICP, CPP, and CBF continues under anesthesia or animal is awaked after removal of monitoring devices. 

*A Direct Infusion into the Cisterna Magna.* This is another frequently used route for blood delivery. It requires significantly more surgical experience but is still relatively easy (for detailed description check references in Table 2). In short, an animal is anesthetized and placed in a prone positioning with the head tilted forward. The atlantooccipital membrane is exposed via a skin incision from the midline on the back of the neck and a delicate dissection of muscles from the occipital bone and C1-2 vertebral bodies. The atlantooccipital membrane is then punctured with a 27-gauge needle or PE-10 catheter that is attached to a syringe filled with fresh autologous blood. The injection follows the same parameters as the percutaneous infusion. The muscle is reapproached with sutures and wound closed and covered with antibiotic creams to speed the healing and prevent infection. Advantage of this approach is a possibility of sealing the hole by tissue glue as the needle is removed.

*A Direct Anterior Intracisternal (Prechiasmatic) Blood Injection*. This route was traditionally used in large animals (dog, monkey) [90, 108] and has been recently adapted to rodents [109–111]. The technique used requires advanced surgical skills, stereotactic apparatus, and access to radiological equipment. It can be achieved via intra(peri)orbital approach [110, 111] with or without enucleation [90] or through transparenchymal approach (Table 2) [109]. Approach used to access prechiasmatic cistern differs among species. In rodents a prechiasmatic cistern is usually approached by placing the animal in prone position and advancing a 27-gauge needle attached to a 1 mL syringe with nonheparin blood stereotactically until the tip reaches the base of the skull and a proper placement in a prechiasmatic cistern is confirmed by flow of CSF into the syringe [109]. The orbit and the optic foramen have also been used to access perichiasmatic cistern.

#### 6.1.2. Blood Injection Parameters: Volume, Length, and Speed

The volume, length, and speed of blood infusion dictate the degree of ICP rise and CPP reduction and thus the intensity of SAH being created. To ensure similarity of SAH intensity within an experimental group these parameters need to be standardized and closely monitored. This however is not a simple task, as intracranial volume differs within and between species making an investigator's control on the injury intensity difficult. Consequently, in current practice a consensus on the injection parameters that work the best in a particular species does not exist, and a wide array of volume, length, and speed options are available and used for injecting blood in a single species. A downfall of this is that since the intensity of SAH depends upon the volume, length, and speed of injection, variations in these parameters makes a comparison and interpretation of results from different laboratories difficult, if not impossible. For instance, SAH in rat has been induced by injecting 100 microL autologous blood over seconds [110], 0.2 mL of blood for more than 1 min [109] or 0.3 mL blood for 1 min [111].

*On the Model*. In a modified aSAH model injection parameters should be guided by the changes of ICP and CPP. The parameters that evoke dramatic but transient reduction in CPP to near zero should be selected and used for creating SAH. These parameters will of course differ between species and even within a species, but the selection criteria (transient reduction in the CPP to near zero) will remain same. This will facilitate comparison data obtained in different laboratories and among different species.

#### 6.1.3. Factors Influencing Choice of Technique Used for SAH Induction

A number of factors need to be considered before a technique can be selected for creating aSAH. Some of these factors are as follows:

- *Simplicity and Reproducibility.* A technique that is simple and reproducible is increasingly attractive and has greater chances of becoming a favored method for studying a problem. A simple technique allows for a short training period and reduces the chances of surgeon's mistake. Reproducibility of injury decreases the cost of a project by reducing the number of animal required for an experiment. Above, we have examined simplicity and reproducibility of available aSAH techniques.
- *Ease of Adaptation.* A technique allowing for adaptation in several animal species permits comparison of results. Several animal species have been used to study SAH. These range from smaller animals: mice, rats, rabbits, and gerbils, to larger animals: cats, dogs, pigs, and nonhuman primates. For animal species used for a particular SAH technique see Table 2 and associated references. Primates, due to their higher ranking in the evolution ladder, are considered the best choice for replication of human conditions. However, not every investigator and laboratory is equipped to use primates. Fortunately, the success of a rabbit embolic model versus failure of favored primate model proves that it is the disease modeling and not the closeness of species to human that translates into a successful treatment. Replication and cross-validation of results in more than one animal species are perhaps a stronger indication of future successful translation in clinical trial. Such option will only be available if the technique used to create SAH is applicable in other species with no or only minimal modifications.
- *Low Mortality with Ethically Acceptable Morbidity*. Since computer simulation cannot be used to study mechanisms and test therapies, animal research remains to be the cornerstone of scientific research and drug development. However, respect for lives of all creatures is essential and is an important consideration in animal research. Reducing distress and suffering in animals is a crucial consideration in development of an animal model. A number of steps can be taken to prevent unnecessary animal suffering during experimentation. These steps include (1) use of perioperative and postoperative analgesia and anesthesia; (2) use of proper life support; (3) aseptic surgical technique; and (4) little amount of surgical manipulation etc.

Use of perioperative and postoperative analgesia and anesthesia during surgery reduce distress caused by the surgical manipulations for inducing SAH. The type and dose of anesthetic and analgesic depends upon the animal species being used. An investigator can refer to species-specific guideline on anesthesia and analgesia provided by their institution for agents that work best in the species used. The depth of anesthesia ensures that animal does not feel pain during surgery. A frequent check of corneal reflex and limb pinch as well as monitoring of heart rate is commonly employed to confirm anesthesia depth. Such as for rat Ketamine-Xylazine combination (50 mg/5 mg/Kg; intraperitoneal administration) is often used for reducing perioperative pain and buprenorphine (0.05 mg/Kg, subcutaneous administration) twice daily for reducing postoperative pain. In addition, inspired isoflurane (1% to 2% in oxygen-supplemented room air) is frequently used during surgery to maintain deep sedation in rats. 

Proper life support during surgery reduces animal mortality. This support includes monitoring and regulation of breathing, body temperature, and a fluid intake. The increase in ICP upon blood infusion may increase pressure at the respiratory centers to the point that animal stops breathing. A respiratory support that ensures breathing such as intubation or placement of a nose cone ensures that animal does not expire. Similarly, unless a project is studying the effect of temperature on injury, body temperature of animal is maintained at 37°C (such as by a thermoblanket) from the start of anesthesia until the animal recovers. For proper hydration ringer lactate is administered as required. 

Aseptic surgical technique protects against infection. As a minimum requirement, this includes sterilization of surgical equipments, applying antiseptics such as iodine to the wounds upon closing and if the project permits, administration of antibiotics to prevent infection from occurring and speed healing.

The amount of surgical manipulation can result into animal death. In general, the more the surgical steps, the more invasive the procedure becomes. In contrast, a simple procedure reduces unnecessary pain and suffering.

*On the Model*. The technique used for SAH induction should be simple, reproducible and allow adaption into different species.

### 6.2. Monitoring of SAH Physiology

Physiological monitoring is an essential feature of modified aSAH model as it confirms the intensity of SAH. This information can be used to ensure that all animals within and across an experimental group receive similar intensity and to interpret the results from different laboratories.

#### 6.2.1. ICP and CPP Changes

Equilibrium between brain, and cranial vault volume via controlled intracranial blood and CSF flow is essential for maintenance of normal ICP. This equilibrium is disturbed by blood released upon aneurysm rupture. An ICP rise that occurs at aSAH reflects subarachnoid blood volume, status of brain and cerebrovascular disturbances. Furthermore, peak ICP value and the pattern of its decline associate with the intensity of injury after SAH [7, 112]. Hence, continuous and reliable ICP monitoring via a simple and easy technique is desired to determine and control the injury intensity and understand the underlying pathophysiologic events after aSAH. 

*ICP Measurement*. Symptoms like headache, nausea, vomiting (particularly projecting), and the presence of papilledema strongly suggest an increased intracranial pressure; however, they do not allow for close monitoring of ICP changes. Fortunately, ICP can be assessed by a number of ways; however all these methods are invasive.

- *Intraventricular Catheter*. In this method a burr hole is drilled in the frontal region, and under either stereotactic or under radiographic guidance a catheter is introduced into the frontal horn or the lateral ventricle and secured to the skin. This method allows for continuous and accurate assessment of ICP and for eventual intervention if an ICP increase jeopardizes CBF.
- *Intraparenchymal Probe.* The placement of an *intraparenchymal probe* with a pressure sensor or a fiber-optic catheter is an alternative to the ventricular catheter. However, this method is prone to a reference drift while recalibration is impossible after the probe is in place. Furthermore, the local changes of pressure evoked by metabolic changes related to disease or (a traumatic probe placement) can dramatically influence recordings.
- *Subdural Bolt.* A burr hole is drilled, and a hollow screw is inserted through the dura, and pulsations of CSF in a subarachnoid space are recorded via a sensor.
- *Epidural Sensor.* A burr hole is drilled, and an epidural sensor is inserted between the skull and the dura to register dural tension (pulsations).

The accuracy of measurements by subdural bolt or epidural sensor is lower than those by intraventricular catheter. Additional caveats are (1) ICP is not uniformly distributed through the brain, and (2) local pressure measurements made by an intraparenchymal probe may not match the intraventricular pressure [113].

*On the Model*. The intraventricular measurement, despite being technically demanding, seems to be a method of choice for the new aSAH model. 

#### 6.2.2. Blood Pressure and Heart Rate Changes (“Cushing's Reflex”)

Cerebral perfusion pressure (CPP) is an important, if not crucial, clinical tool that provides information on perfusion of brain [113]. CPP falls as ICP increases. An ICP rise that is near or above systolic blood pressure leads to complete perfusion arrest; a reduction of CPP to zero. Recovery of CPP begins as ICP declines after reaching a peak. CPP is estimated as the difference between ICP and mean arterial blood pressure: CPP = MABP − ICP. 

Furthermore, an increase in ICP at SAH evokes Cushing's reflex, a hypothalamic response to ischemia. During this reflex systolic blood pressure rises, heart rate decreases, and respiration becomes irregular (sympathetic stimulation); each either directly or indirectly influences CPP and CBF. Thus, monitoring of BP and heart rate changes is necessary to access CPP changes after SAH.

*(1) Blood Pressure Measurement*. Mean arterial pressure can be measured by invasive and noninvasive methods.

- *Invasive Method*. This surgical method is based on experiments conducted by Stephen Hales in 1733, that showed that blood pressure and heart beat can be observed by a glass tube inserted into an artery of horse who inserted a glass tube in artery of horse and observed changes in blood pressure with the heart beat [114]. Not much has changed since then, and to obtain reliable and long-lasting monitoring in surgical settings under anesthesia, a sterile catheter is placed into radial or femoral artery. This method is used mostly for acute experiments and/or in bigger animals but has been used to measure blood pressure in small animals: rabbit (ear) and rodents (tail artery).
- *Noninvasive Method.* This method is further divided into auscultatory or oscillometric methods.

The *auscultatory *method is most commonly used for measuring blood pressure in clinics. It is based on Korotkoff's 1905 discovery of the auscultatory sounds [115]. This method uses a blood pressure cuff and stethoscope (or more recently a microphone), which are applied on the arm (monkey), leg, or tail (rodents) to register animal's pulse tones. It allows for single, serial, or continuous measurements but usually requires anesthesia, which may influence the results. Moreover, if the stethoscope is used, results can be inconsistent and operator dependent. However, the measurements of systolic and diastolic pressures allow for an easy and often automated assessment of mean arterial pressure.

The *oscillometric method* is widely used for blood pressure measurement in the experimental settings. It measures oscillations caused by blood flow (i.e., pulse) by means of a pressure cuff. This simple method does not require a skilled operator and hence can be automated for blood pressure recording. However, it does have several, above-mentioned, limitations related to the use of a cuff.

*(2) Heart Rate Monitoring*. Sympathetic stimulation during Cushing reflex leads to reduction in heart rate (bradycardia) and significant increase of BP. The following techniques have been used for monitoring heart rate and other cardiac changes following SAH.

- *ECG Monitoring*. ECG changes are registered when the ICP increases toward the systolic arterial pressure.
- *Transesophageal Echocardiography*. Can be used in large animals to assess wall motion changes and aortic and pulmonary flow velocities at SAH [116].
- *Serum Markers of Myocardial Injury. *An increased serum creatine kinase-MB and cardiac troponin-1 (cTn-1) concentration is often used to diagnose acute myocardial injury after SAH. However, as CK-MB can be released from non-cardiac muscle damage, cTn-1 is a superior indicator of myocardial injury [117].

*On the Model*. In addition to the ICP measurements, BP monitoring is a required feature of a modified aSAH animal model. The technique used for monitoring BP and cardiac changes in the new aSAH animal model will depend upon nature of experiment and its requirements. If an animal survival is required, then noninvasive BP monitoring should be used. Similarly, if the effect of SAH on heart rate is of concern, then a simple ECG monitoring will work fine.

#### 6.2.3. CBF Changes and Possibility of Repeated Arteriography or TCD for a Delayed Vasospasm Assessment

CBF monitoring and vasospasm assessment provide useful tools to examine potential therapeutic options. An animal model provides these assessments and, in addition, can help establish the influence of acute phase on the following subacute and delayed phases of brain injury after aSAH. 

*CBF Monitoring*. CBF can be assessed quantitatively or qualitatively. ^133^ *xenon method* is a method for quantitative assessment of CBF, which was described by Kety-Schmidt [118]. CBF is calculated from data obtained from several detectors placed on the head surface after administration of radioactive xenon gas. This method is widely used in both clinical and experimental settings. However, it measures CBF mostly from cortical and subcortical structures of the middle cerebral artery, and the measurements obtained are not reproducible by other CBF measurement methods. In addition, this method is cumbersome, requires significant investment, knowledge, and experience.

- *Thermal Diffusion Method. *This method estimates cortical or interstitial blood flow from the temperature difference between the two gold plates at the tip of the probe placed on or in the brain through a burr hole [119–121]. This method provides continuous quantitative real-time CBF. However, measurements are made from a limited (local) area only and may not represent the whole brain (global) CBF changes.
- *Transcranial Doppler Method* (TCD). This is a noninvasive method that was introduced by Aaslid et al. in 1982 [122]. It measures blood flow velocity and not blood flow. The linear relationship between CBF and mean flow velocity under most of the experimental and many clinical conditions allows for accurate assessment of CBF by TCD method and permits real-time CBF measurements [121–123]. This method is easy to use, allows for continuous data collection over a long period of time, can be used repeatedly, and allows comparison with other experiments or data sets [113, 123]. The usefulness of TCD for assessment of CBF and arterial diameter has been confirmed by numerous experimental and clinical studies of SAH [121, 124–128]. In addition, TCD assesses vascular resistance and reactivity as well as status of autoregulation of CBF. This is of significant value since CBF is constant in the CPP range of about 50–150 mmHg because of autoregulation, which is frequently disturbed after aSAH. The limitations of TCD include indirect CBF measurement and inter-operator variability.
- *Jugular Oximetry. * As TCD, *jugular oximetry* does not measure CBF directly. Here, CBF is calculated from arteriojugular oxygen saturation difference (AJDO_2_). The measurements assess CBF in relation to metabolic activity but are adequate only if coupling between CBF and metabolism is intact. Another limitation is that oximetry assesses oxygen content in a jugular bulb that may better represent hemispherical and not global CBF.
- *Cerebral Angiography.* Spasm in large cerebral arteries sets in 3–7 days after SAH. Angiography is frequently used to assess the presence and severity of delayed arterial vasospasm. Though this technique is invasive it can be used repeatedly to follow the development and effects of pharmacological intervention on the delayed vasospasm [129].
- *EEG Monitoring.* EEG changes are registered when the ICP surpassed the systolic arterial pressure and the electrical silence results of arrest of the cerebral circulation.

*On the Model*. CBF measurement is crucial for a modified aSAH model and should be performed using a technique that is reliable, simple, easy, noninvasive, and allows repeated measurements. TCD fulfills this selection criterion.

### 6.3. Outcome Assessment

An animal outcome is an essential endpoint of an aSAH study. It confirms the importance of a pathway being studied in aSAH induced injury and helps decide whether modification of this pathway would be beneficial. It is also essential that outcome assessments studied in animals are relevant to the human condition so that treatments found effective in animals can be translated to the patients [99]. 

In aSAH patient neurological and functional deficits develop early and/or after several days. In patients, status at admission and early deficits are assessed by the Hunt and Hess, the Glasgow coma (GCS), and the World Federation of Neurological Surgeons (WFNS) grading scales [130]. The long-term outcome in SAH patients is assessed by Katzman, Rankin, and/or Barthel scores. In animals, neurological injury is studied *indirectly *as diminished response to an external stimulus or reduced function or *directly* as death of brain cells by immunostaining or assays for apoptosis, autophagy, or neurodegeneration. The methods used for assessing neurological and functional deficits in SAH animals are less than perfect and often erroneously incorporate procedures intended for assessing focal ischemic injury. Furthermore, though a battery of exams for a number of species exists, a species-specific limitation for assessments is often not recognized (for review see [131, 132]). The review by Jeon and colleagues provides an excellent guide to the techniques used to assess outcome in rodents after aSAH [99].

*On the Model*. A new aSAH animal model should induce consistent and reproducible immediate-gradual and transient-permanent injury and deficits. Thus, it should use scales and exams for injury assessment that are similar or equivalent to the ones used in SAH patients. This strategy will increase the chances of successful translation of a therapy found beneficial in animals.

## 7. Modified aSAH Model

We applied quite a few restrictions to establish an improved aSAH model and and came up with several must have essentials and a spectrum of choices rather than a single, one-fits-all solution. An investigator of course will select the technique that suits the animal species and the phase of injury (acute versus delayed) being studied and permitted by the laboratory environment. The approach that in our opinion will work the best is as follows.

1. Blood is injected, so that it pools in the subarachnoid space and elevates ICP to a level that CPP reduces to zero creating TGI.
2. Physiological parameters that change after SAH and associate with the intensity of injury are monitored:
  - early ICP change via an intraventricular catheter;
  - early BP change via an oscillometric method;
  - early CBF change via TCD;
  - delayed vasospasm via repeated arteriography or TCD.
3. Outcome assessments are made using scale and exams that are equivalent to the ones used for assessing clinical outcome.
4. Additional attributes are adaptable to other species (range from rodents to primates) with little modification and low mortality and morbidity.

## 8. Summary

Inadequate disease modeling may have contributed to the failure of improving outcome in aSAH patients. We presented here a proposal of a modified model of aSAH that incorporates all of the components and elements of injury after aSAH, which may provide a better resource for studying the injury mechanisms and developing a treatment.

## Figures and Tables

**Table:** (a) Complete TGI models

TGI method	Key features	Species	References
Cardiac arrest (i) KCl injection	Epinephrine injection, defibrillation, and CPR are used for resuscitation	Mouse, rat and monkey	[10–12]
(ii) Ventricular fibrillation	Can be used with CPR to study resuscitation	Cat, dog, pig and monkey	[13–16]

Aortic occlusion	Inhibits flow throughout the body	Rat, rabbit, cat and dog	[17–19]

Neck cuff/tourniquet with hypotension	Inhibition of blood flow to the head	Rat, cat, dog and monkey	[20–24]

Extracranial artery occlusion			
(i) Innominate and subclavian arteries	Inhibition of blood flow to the head	Cat	[25, 26]
(ii) Brachiocephalic and subclavian near aortic origin		Monkey	[27]

**Table:** (b) Incomplete TGI models

TGI method	Key features	Species	References
Intracranial hypertension			
(i) Fluid infusion in cerebral cistern	A brain compression injury	Rabbit, cat, dog and monkey	[28–30]
(ii) Balloon inflation		Rat, cat, dog and monkey	[31–34]

Extracranial artery occlusion	Immediate ischemia and reperfusion allows possibility of permanent occlusion		
Bilateral common carotid (2-VO)			
(i) Without hypotension	Creates mild-to-moderate injury	Mouse, rat, gerbil, sheep and monkey	[35–39]
(ii) With hypotension		Rat, rabbit, cat and monkey	[40–42]
Bilateral common carotid + vertebral arteries (4-VO)	Creates severe injury	Rat, rabbit, cat, dog and monkey	[43–48]

**Table 2:** Experimental models of aSAH and/or vasospasm.

Species	SAH method				Phase studied		Reference
	Artery puncture	Blood injection		Clot	EBI	Vasospasm	
		Single	Double				
Mouse	+	+	+	−	+	?	[53–55]
Rat	+	+	+	−	+	+/−	[56–60]
Rabbit	+	+	+	−	+	+/−	[61–63]
Cat	+	+	+		+		[5, 64–66]
Pig		+	+	+	+	+	[67, 68]
Dog	+	+	+	+	+	+	[69–73]
Nonhuman primate	+	+	+	+	+/−	+	[74–77]

**Table 3:** Risk factors of TGI versus aneurysmal SAH.

Factor	TGI	aSAH
High blood pressure	Shared	Shared
Smoking	Shared	Shared
Alcohol abuse	Shared	Shared
Stress	Shared	Shared
Cardiac arrest or shock that creates prolonged hypoxia or hypoglycemia	Stroke only	
Pathologically elevated cerebral metabolic rate	Stroke only	
Decreased cerebral perfusion pressure	Stroke only	
Age (years)	≥65	≤56
Gender	Men prevalence	Women prevalence
Intracranial aneurysm	−/+	+
